# Whey Protein Supplementation Improves the Glycemic Response and May Reduce Non-Alcoholic Fatty Liver Disease Related Biomarkers in Women with Polycystic Ovary Syndrome (PCOS)

**DOI:** 10.3390/nu13072451

**Published:** 2021-07-17

**Authors:** Emily L. Zumbro, Manisha Rao, Shenavia Balcom-Luker, K. Shane Broughton, Monique J. LeMieux

**Affiliations:** 1 School of Health Promotion & Kinesiology, Texas Woman’s University, Denton, TX 76204, USA; elzumbro@uab.edu (E.L.Z.); manisharao1210@gmail.com (M.R.); 2 Nutrition & Food Sciences, Texas Woman’s University, Denton, TX 76204, USA; sbalcom@twu.edu (S.B.-L.); kbroughton@twu.edu (K.S.B.)

**Keywords:** polycystic ovary syndrome, sex hormone-binding globulin, non-alcoholic fatty liver disease, whey proteins, blood glucose, exploratory study

## Abstract

Polycystic ovary syndrome (PCOS) increases type 2 diabetes and non-alcoholic fatty liver disease (NAFLD) with insulin resistance. We hypothesized that a 35 g whey preload would improve insulin sensitivity and glucose handling while reducing biomarkers associated with NAFLD. Twenty-nine age-matched women (CON = 15, PCOS = 14) completed oral glycemic tolerance tests following baseline (Day 0) as well as an acute (Day 1) and short-term whey supplementation (Day 7). Whey had an interaction effect on glucose (*p* = 0.02) and insulin (*p* = 0.03), with glucose remaining stable and insulin increasing with whey supplementation. Insulin sensitivity (*p* < 0.01) improved with whey associated with increased glucagon secretion (*p* < 0.01). Alanine aminotransferase (ALT), and aspartate aminotransferase (AST) remained unchanged, but “day” had an effect on the AST:ALT ratio (*p* = 0.04), whereas triglycerides and sex hormone binding globulin overall were greater in the PCOS group (*p* < 0.05). Total cholesterol decreased in PCOS (by 13%) and CON (by 8%) (NS). HepG2 cells treated with plasma from participants before and after whey decreased lipid accumulation in the PCOS group after whey (*p* < 0.05). Whey provided an insulinogenic and glycemic homeostatic effect in women with PCOS with the potential to combat NAFLD-consequences.

## 1. Introduction

Polycystic ovary syndrome (PCOS) affects 6–9% of premenopausal women worldwide [1] and is characterized by metabolic dysfunction, hyperandrogenism, hirsutism (HS), polycystic ovaries, chronic anovulation, and infertility [2,3,4]. Insulin resistance (IR) and the consequential impaired glucose tolerance and hyperinsulinemia are common metabolic idiosyncrasies exhibited by polycystic women [4]. Obesity is common in women with PCOS, contributing to metabolic syndrome in conjunction with IR, increasing a woman’s risk for cardiovascular disease [5,6], type 2 diabetes (T2DM) [7,8], and non-alcoholic fatty liver disease (NAFLD) [9,10,11].

High insulin levels characteristic of IR result in suppressed hepatic secretion of sex hormone binding globulin (SHBG) [12,13,14], increased glucagon secretion [15], elevated alanine aminotransferase (ALT) [16], and potential elevation of aspartate aminotransferase (AST). In a fasted state, low SHBG and elevated ALT and glucagon concentrations are associated with PCOS [17,18,19,20].

NAFLD is characterized by excess fat and cholesterol accumulation in the liver in individuals with little to no alcohol consumption [21]. It includes hepatic steatosis, defined by intracellular accumulation of triglyceride (TG) in the liver, and may progress to non-alcoholic steatohepatitis (NASH), fibrosis, cirrhosis, and hepatocellular carcinoma. Elevated ALT blood levels is also a common index for NAFLD with a AST-to-ALT-ratio (AST:ALT) < 0.8, indicating positive for hepatic steatosis [22] where circulating ALT concentration exceeds circulating AST concentration. The current hypothesis for the cause of elevated aminotransferases is two-fold: (1) ALT activity is upregulated due to the shift towards gluconeogenesis as a consequence of IR and (2) induction of oxidative stress through the accumulation of reactive oxygen species resulting in liver inflammation and reduced hepatocyte integrity [23,24]. It is thought that the combination of these events leads to elevations of aminotransferases in circulation and ultimately contribute to NAFLD development.

Hyperandrogenism has been associated with elevated ALT activity in women with PCOS, suggesting that excess androgens may have a harmful effect on the liver in this population [25]. Women with PCOS have an increased prevalence of NAFLD and are more likely to develop more severe stages of the disease such as NASH, cirrhosis and hepatocellular adenoma [26,27,28]. Women with PCOS have decreased cholesterol efflux capacity, which has been shown to be an independent predictor of subclinical cardiovascular disease [29]. Dysregulated cholesterol metabolism has also been demonstrated in patients with NAFLD. Those with more severe forms of NAFLD synthesize greater amounts of endogenous cholesterol but are unable to properly remove cholesterol from the endogenous pool in the liver, thus causing its accumulation [30]. Similarly, the dyslipidemia and hypercholesterolemia associated with PCOS are likely due to alterations in cholesterol metabolism as the same pathways are utilized.

Women with PCOS are encouraged to lose weight if overweight or obese and maintain an optimal weight through diet and exercise. Weight loss in women with PCOS can result in an improved metabolic profile leading to a decreased incidence of metabolic syndrome and T2DM. Caloric restriction is a primary nutritional intervention used for weight loss and improvement of metabolic health [31,32]. Diets high in protein with a calorie restriction are common recommendations due to their satiety effects and preservation of lean body mass with weight loss [33].

Whey protein supplementation is a caloric addition and substitution to the diet that increases essential amino acid intake, stimulates satiety, reduces inflammation, and exerts a post-prandial insulinogenic effect [34,35,36]. Whey protein has been established as an appropriate means to promote metabolic control in overweight, obese, and metabolic disease populations [34,35,36,37,38]. Few studies, however, have examined the effect of whey protein on similar parameters in women with PCOS. Additionally, whey protein may be beneficial in reducing ALT and AST concentrations [39,40]. Due to the nature of PCOS and the increased risk for the development of T2DM and other metabolic anomalies, the purpose of this exploratory study was to compare the hormonal and liver enzyme response to a single dose and 7-day supplementation of 35 g whey protein isolate (WPI) preload in an oral glycemic tolerance test (OGTT) in women with PCOS and healthy controls. We hypothesize that a WPI preload will improve insulin sensitivity and glucose handling in women with PCOS while reducing biomarkers associated with NAFLD.

## 2. Materials and Methods

### 2.1. Participants

A total of 75 participants were recruited for this study within the university community from October 2017 to April 2019. Of these, four volunteers dropped out due to gastrointestinal side effects (nausea, vomiting and diarrhea) of the OGTT beverage or WPI while 42 discontinued due to scheduling conflicts. A total of 29 women completed the study and were included in analyses. They comprised of 14 medically diagnosed PCOS women based on the Rotterdam criteria [41] (PCOS; age = 22.9 ± 5.8 year; body mass index (BMI) = 33.7 ± 9.5 kg/m^2^) and 15 premenopausal controls (CON; age = 21.1 ± 3.2 year; BMI = 24.4 ± 4.0 kg/m^2^). The Rotterdam criteria consist of chronic anovulation (less than 9 menstrual cycles within the past 12 month) along with clinical hyperandrogenemia and/or polycystic ovarian morphology as indicated by ultrasound. Exclusion criteria were as follows: any medical diagnosis other than PCOS, use of medication for T2DM treatment, participating in regular exercise (i.e., ≥30 min/day, ≥3 days/week), utilizing a specialized diet, or engaged in any assisted reproductive programs. None of the participants who completed the study were on estrogen-containing contraception.

### 2.2. Study Design

Preliminary screening and informed consent occurred at recruitment to assess exclusion criteria along with physical assessments. Weight (kg), height (cm), BMI (kg/m^2^), waist-to-hip ratio (WHR), body fat percentage (BF%), and lean body mass (LBM) were recorded during the preliminary screening visit as baseline data. HS and acanthosis nigricans (AN) screening were performed on all participants during preliminary screening and post-intervention. HS was self-assessed using the modified Ferriman Galleway (mFG) scale, whereas AN was assessed by a single researcher throughout the study. AN scores were determined based on severity and texture at the following sites: neck severity, axilla severity, neck texture, knuckles, elbows, and knees. Body composition for BF% and LBM was measured during preliminary screening via dual x-ray absorptiometry (DXA; Lunar Prodigy, General Electric Healthcare, Little Chalfont, UK). Participants were instructed to partake in regular eating patterns and recorded a 3-day dietary record via MyFitnessPal (Under Armour ^®^, Inc., San Francisco, CA, USA) prior to intervention to establish average total caloric daily intake. No differences between groups were observed (Table 1). Participants were advised to refrain from altering their physical activity during the course of the study. Briefly, testing for participants occurred on three separate visits: an oral glycemic tolerance test (OGTT) with a 250 mL water preload followed by a 75 g glucose load (Tru-Glu 100, Fischer Scientific™, Pittsburg, PA, USA) taking place during the early follicular phase of the menstrual cycle (Day 0; baseline); an OGTT with a 35 g WPI (35 g WPI, 76.58 mg sucralose and 975.09 mg vanilla or chocolate flavor, Glanbia©, Chicago, IL, USA) preload in 250 mL of water followed by a 75 g glucose load during the early follicular phase of the next menstrual cycle (Day 1); and an OGTT with a 35 g WPI preload in 250 mL of water on the seventh day following the Day 1 test (Day 7). To minimize the hormonal impact, the early follicular phase of the menstrual cycle was selected as the timepoint for evaluation. Various dietary components can stimulate insulin release differentially, as such, it was decided to use 250 mL of water as the non-caloric baseline. The OGTT testing occurred following a 10 h overnight fast. The 250 mL water only and 35 g WPI preload was administered 30 min prior to the 75 g OGTT for each respective test, with each one ingested within 5 min. Following evaluation of previous studies [42,43,44,45,46] and discussions with the protein provider, 35 g was identified as the upper tolerable limit that did not have gastrointestinal issues. Participants consumed 35 g of flavored (chocolate or vanilla) WPI 30 min prior to lunch daily between Day 1 and Day 7 of testing and were given the option to add additional artificial flavoring on non-testing days to optimize intervention adherence. Participants were requested to avoid skipping breakfast, continue consuming typical meals ad libitum, to not participate in any exercise programs during the study, and to consume the same meal on the night before each of their OGTTs.

### 2.3. Blood Sampling

**Figure 1:**
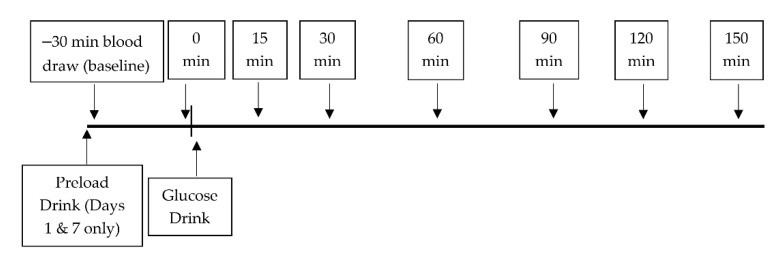
OGTT study design and blood sampling timepoints for day 0, 1 and 7.

Figure 1 outlines the timeline for blood sampling. Venous blood samples were acquired at −30/pre-preload, 0/pre-glucose load, 15, 30, 60, 90, 120, and 150 min on each testing day. EDTA and BDTM P800 with protease, esterase, and DPP−4 inhibitors vacutainers (BD Biosciences, San Jose, CA, USA) were used to collect whole blood samples for each timepoint. An additional tube without an anticoagulant was obtained for each testing day at the −30 min timepoint for subsequent serum isolation (BD Biosciences, San Jose, CA, USA). EDTA collected plasma samples were used for the analyses of glucose, insulin, triglyceride (TG), total cholesterol (TC), ALT, and AST. BDTM P800 collected plasma samples were used for the analysis of glucagon. Serum samples were used for the analysis of SHBG. Plasma and serum were isolated by centrifugation at 3000 rpm for 15 min with aliquots stored at −80 °C for subsequent analyses. Plasma and serum samples were used for biochemical analyses.

### 2.4. Biochemical Analyses

Glucose, insulin, and glucagon were analyzed across all timepoints on each treatment day, whereas ALT, and AST, were analyzed only at the −30 min timepoint for each treatment day. SHBG, TC and TG were analyzed only at the −30 min timepoint for days 0 and 7 only. Plasma glucose, ALT, and AST were analyzed using a Biolis 24i/CLC480 automated spectrophotometer (Carolina Liquid Chemistries Corporation, Winston-Salem, NC, USA). TG, ALT and AST activities (ALTact and ASTact, respectively) were measured using a colorimetric activity assay from Cayman Chemicals (Ann Arbor, Michigan). An ELISA assay (ALPCO, Salem, NH, USA) was used to analyze plasma insulin. Plasma glucagon was analyzed via a human metabolic hormone multiplex assay (EMD Millipore ™ Corporation, Billerica, MA, USA). A separate ELISA assay (Eagle Biosciences, Inc., Nashua, NH, USA) was used to assess SHBG serum concentrations. TC was measured using the Cholesterol LiquiColor ^®^ enzymatic test (Stanbio Chemistry, Boerne, TX, USA). All samples were analyzed in duplicate.

### 2.5. HepG2 Lipid Accumulation

HepG2 cells were seeded into 6-well (30,000/cm^2^) plates for lipid accumulation assays. The cells were grown to confluence using Dulbec47co’s Modified Eagle Medium (DMEM) low glucose media (Gibco/Thermo Fisher Scientific, Waltham, MA, USA), 1% Penicillin-Streptomycin-Neomycin Antibiotic Mixture (PSN, Thermo Fisher Scientific), and fetal bovine serum (FBS, Thermo Fisher Scientific). Then the cells were switched to an FBS-free media (DMEM and PSN only) for 48 h, followed by treatment of FBS-free media with 1% human plasma sample (PCOS Day 0 (PCOS-D0), PCOS Day 7 (PCOS-D7), CON Day 0 (CON-D0) or CON Day 7 (CON-D7)) added for an additional 48 h. Plasma samples added were from baseline draws on the respective days with a minimum of 18 h post previous ingestion of WPI supplementation. Plasma from 6 different participants from each group of the clinical part of this study were used for these experiments (one plasma source per well). Once HepG2 cells had been treated with human plasma for 48 h, lipid accumulation was measured using AdipoRed™ Assay Reagent (Lonza, Walkersville, MD, USA) per manufacturer’s instructions for a 6-well plate [47].

### 2.6. Calculations

Matsuda index is an assessment of insulin sensitivity from the OGTT that provides an approximation of whole-body insulin sensitivity (WBISI). The formula for the calculation is 10,000/√ (fasting glucose × fasting insulin) (mean glucose × mean insulin) [48].

The Oral Disposition Index (DI_O_) is a novel assessment that provides a measure of β-cell function adjusted for insulin sensitivity and is predictive of development of diabetes over 10 years. The formula for the calculation is the change in insulin concentrations within the first 30 min (ΔI_0–30_)/change in glucose concentrations within the first 30 min (ΔG_0–30_) × 1/fasting insulin [49]. For this study, we looked at the first 30 min after WPI ingestion as well as the first 30 min after glucose ingestion. For the WPI alone calculations, 13 subjects (8 PCOS and 5 CON) were excluded because of negative or zero ΔI_0–30_/ΔG_0–30_, whereas 11 subjects (3 PCOS and 8 CON) were excluded after glucose ingestion.

### 2.7. Statistics

Data are expressed as mean ± standard error of the mean (SE), with means compared across analyses using SPSS v25.0 (IBM ™, Armonk, NY, USA). A between subjects multivariate analysis was used to analyze age, BF%, LBM, WHR, and BMI. A mixed model analysis of covariance (ANCOVA) was used to analyze glucose, glucose iAUC, insulin, insulin iAUC, glucagon, glucagon iAUC, TC, TG, SHBG, ALT, AST, AST:ALT, ALTact and ASTact. BF% was used as the covariate for each test. A two-way mixed analysis of variance (ANOVA) was also run to understand the effects of group and day on DI_O_, SHBG, ALT, AST, AST:ALT, ALTact, ASTact, TG, and TC concentrations. A one-way ANOVA was conducted to determine if lipid accumulation in HepG2 cells was different for groups with different plasma sources. Post hoc analysis was performed with a Bonferroni adjustment for the ANCOVAs and two-way mixed ANOVAs and with Tukey for the one-way ANOVA. Statistical significance for all tests was set at *p* ≤ 0.05.

## 3. Results

### 3.1. Participants

Overall, 75 participants were recruited, with a total of 29 participants completing the exploratory study, 14 PCOS participants and 15 age-matched controls. Table 2 summarizes physical characteristics data across groups. Age, height (cm), and waist-to-hip ratio did not differ between groups. All other differences for baseline participant characteristics were significant between groups (*p* < 0.05). Table 3 summarizes AN and HS characteristics between groups at baseline. For AN, both measurements at the neck were different while the axillae, knuckles, elbows, and knees did not differ between groups. For HS, sites for chest, upper back, lower back, upper arm, and lower abdomen were not significant between groups. All other HS measurements were significant between groups (*p* < 0.05).

### 3.2. Glucose and Glucose IAUC

**Figure 2:**
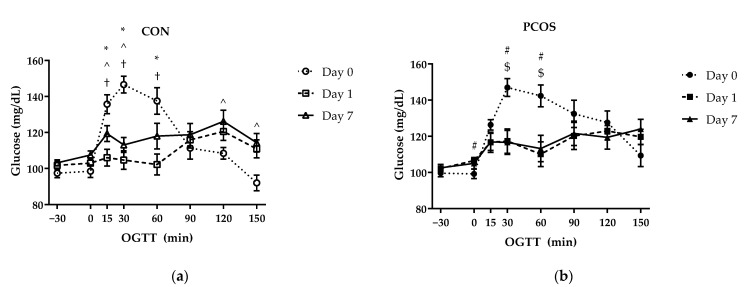
Mean (± SE) glucose concentrations in (**a**) CON (*n* = 15) and (**b**) PCOS (*n* = 14) across timepoints. * = significance between Day 0 and Day 1 for CON; ^ = significance between Day 0 and Day 7 for CON; † = significance between Day 1 and Day 7 for CON; # = significance between Day 0 and Day 1 for PCOS; $ = significance between Day 0 and Day 7 for PCOS, *p* < 0.05. CON = non-PCOS women; OGTT = oral glycemic tolerance test; PCOS = women with polycystic ovarian syndrome.

Glucose results indicated a significant interaction effect of time × day × group [F(14, 364) = 1.999, *p* = 0.02] (Figure 2). Post hoc tests revealed group differences on Day 0 at timepoint 15 min (*p* = 0.02) and on Day 7 at timepoint 60 min (*p* = 0.03), as shown in Figure 2. Additional analyses indicated differences at timepoints within each group between days. For the CON group, Day 0 glucose levels were greater than Day 1 levels at 15 (*p* < 0.01), 30 (*p* < 0.01), and 60 (*p* < 0.01) min; Day 0 glucose levels were greater than Day 7 levels at 15 (*p* = 0.03) and 30 (*p* < 0.01) min and lower than Day 7 levels at 120 (*p* = 0.01) and 150 (*p* = 0.01) min; and Day 1 levels were lower than Day 7 levels at 15 (*p* = 0.02), 30 (*p* = 0.04), and 60 (*p* = 0.01) min. For the PCOS group, Day 0 glucose levels were lower than Day 1 at 0 (*p* = 0.02) min and greater than Day 1 levels at 30 (*p* < 0.01) and 60 (*p* < 0.01) min; and Day 0 glucose levels were greater than Day 7 levels at 30 (*p* < 0.01) and 60 (*p* < 0.01) min. Overall, women with PCOS remained at elevated glucose longer in the day 0 OGTT. While whey blunted the max glucose concentration for both groups, CON appeared to be return to normal by 150 with whey, whereas the PCOS were still on an upward trajectory. This indicates that whey in PCOS may delay circulatory glucose clearance.

**Figure 3:**
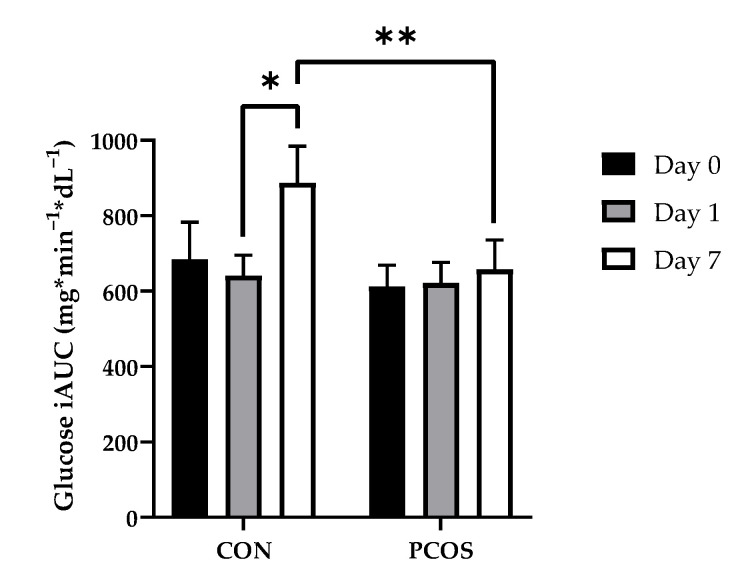
Glucose iAUC mean values (±SE) in CON (*n* = 15) and PCOS (*n* = 14). * = significance between days; ** = significance between groups for day, *p* < 0.05. CON = non-PCOS women; iAUC = incremental area under the curve; PCOS = women with polycystic ovarian syndrome.

Figure 3 displays iAUC values for glucose across all days for both groups. No interaction nor main effects were found for day × group [F(2, 52) = 2.89, *p* = 0.07], day [F(2, 52) = 1.826, *p* = 0.17], nor group [F(1, 26) = 1.74, *p* = 0.20].

### 3.3. Insulin and Insulin IAUC

**Figure 4:**
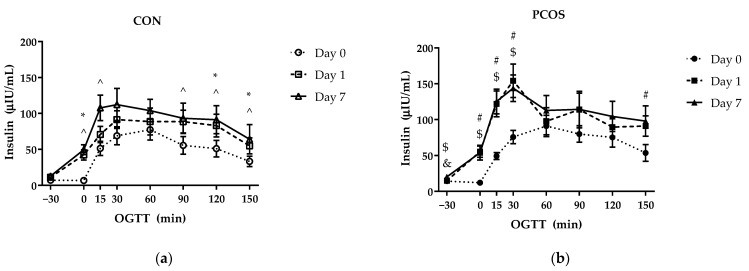
Mean (±SE) insulin concentrations in (**a**) CON (*n* = 15) and (**b**) PCOS (*n* = 14) across timepoints. * = significance between Day 0 and Day 1 for CON; ^ = significance between Day 0 and Day 7 for CON; # = significance between Day 0 and Day 1 for PCOS; $ = significance between Day 0 and Day 7 for PCOS; & = significance between Day 1 and Day 7 for PCOS, *p* < 0.05. CON = non-PCOS women; OGTT = oral glycemic tolerance test; PCOS = women with polycystic ovarian syndrome.

An interaction effect of time × day × group [F(14, 364) = 1.85, *p* = 0.03, ηp^2^ = 0.07] was found for insulin concentrations (Figure 4). Post hoc tests revealed a difference on Day 7 for baseline values between groups (*p* = 0.03), as depicted in Figure 4. Insulin values increased across timepoints 15, 30, 60, 90, and 120 min from baseline values on Day 0 for both the CON and PCOS groups (*p* < 0.05) with insulin concentration remaining elevated in the PCOS group. Additional analyses revealed differences at timepoints within each group between days. For the CON group, Day 0 insulin levels were lower than Day 1 levels at 0 (*p* < 0.01), 120 (*p* = 0.01), and 150 (*p* = 0.02) min; and Day 0 insulin levels were lower than Day 7 levels at 0 (*p* < 0.01), 15 (*p* < 0.01), 90 (*p* = 0.01), 120 (*p* < 0.01) and 150 (*p* = 0.04) min. For the PCOS group, Day 0 insulin levels were lower than Day 1 levels at 0 (*p* < 0.01), 15 (*p* < 0.01), 30 (*p* < 0.01), and 150 (*p* < 0.01) min; Day 0 insulin levels were lower than Day 7 levels at −30 (*p* = 0.01), 0 (*p* < 0.01), 15 (*p* < 0.01), and 30 (*p* < 0.01) min; and Day 1 insulin levels were lower than Day 7 levels at −30 min (*p* = 0.01).

**Figure 5:**
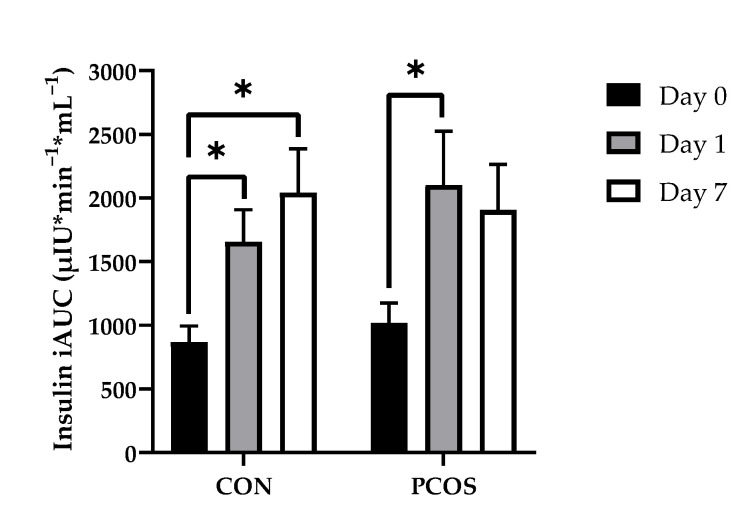
Insulin iAUC mean values (±SE) in CON (*n* = 15) and PCOS (*n* = 14). * = significance between days, *p* < 0.05. CON = non-PCOS women; iAUC = incremental area under the curve; PCOS = women with polycystic ovarian syndrome.

No interaction effect of day × group [F(2, 52) = 2.95, *p* = 0.06] nor main effects for both day [F(2, 52) = 1.27, *p* = 0.29] and group [F(1, 26) = 0.14, *p* = 0.71] were found to impact insulin iAUC values (Figure 5). Post hoc analyses revealed Day 0 insulin iAUC to be lower than Day 1 (*p* = 0.02) and Day 7 (*p* < 0.01) for the CON group, whereas Day 0 was lower than Day 1 only (*p* < 0.01) in the PCOS group.

### 3.4. Insulin Sensitivity

**Figure 6:**
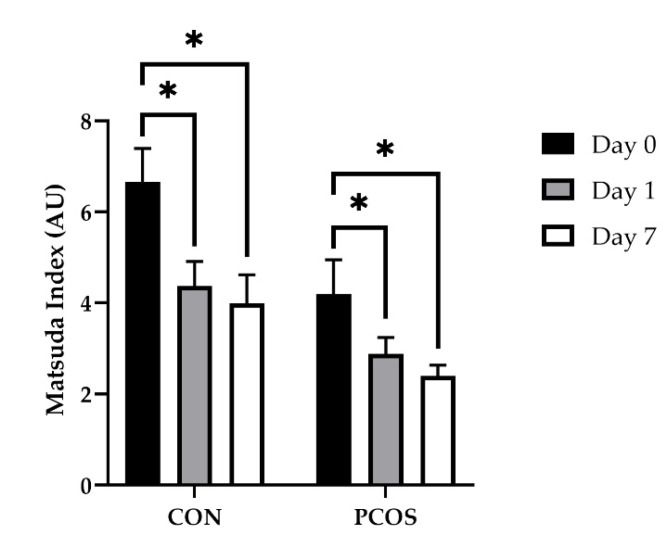
Matsuda index mean values (±SE) in CON (*n* = 15) and PCOS (*n* = 14). * = significance between days, *p* < 0.05. CON = non-PCOS women; PCOS = women with polycystic ovarian syndrome.

Figure 6 displays the Matsuda Index values across all days for both groups. No interaction effects of day × group [F(2, 52) = 0.448, *p* = 0.641] were found. Alternatively, a main effect of day [F(2, 52) = 11.165, *p* < 0.01, ηp^2^ = 0.30] was found with no additional effect of group [F(1, 26) = 1.046, *p* = 0.316]. Post hoc analyses revealed Day 0 values were higher compared to both Day 1 (*p* < 0.01) and Day 7 (*p* < 0.01) overall. More in-depth analyses revealed Day 0 values to be higher compared to both Day 1 (*p* < 0.01) and Day 7 (*p* < 0.01) within both the CON and PCOS groups.

**Figure 7:**
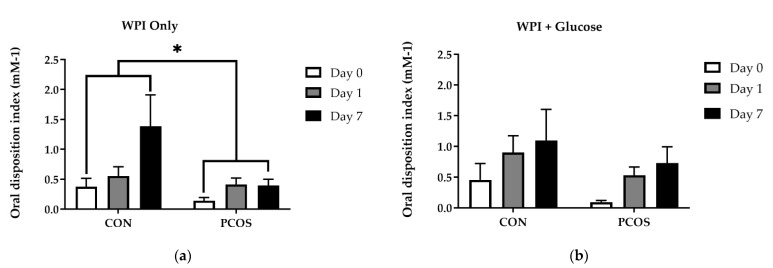
DI_O_ mean values (±SE) in CON (*n* = 15) and PCOS (*n* = 14) 30 min after (**a**) WPI ingestion and (**b**) glucose ingestion. DI_O_ for WPI ingestion only, showed no interaction effect of group and day but did indicate the main effects of group (*p* = 0.028) and day (0.015). DI_O_ after glucose ingestion, yielded no significant interaction or main effects. * = significance between groups, *p* < 0.05. CON = non-PCOS women; DI_O_ = Oral Disposition Index; PCOS = women with polycystic ovarian syndrome.

Figure 7 displays the Oral Disposition Index (DI_O_) values across all days for both groups. DI_O_ after WPI ingestion alone (Figure 7a) showed no interaction effect of day × group [F(1.3, 32) = 3.299, *p* = 0.075]. Alternatively, the main effects of both group [F(1, 16) = 5.823, *p* = 0.028] and day [F(1.3, 32) = 6.273, *p* = 0.015] were found. Post hoc analyses revealed CON values were higher compared to PCOS values overall and Day 7 values were higher than Day 0 values overall (*p* = 0.033). After glucose was ingested (Figure 7b), DI_O_ yielded no interaction effect of day × group [F(2, 30) = 0.000, *p* = 1.000], nor main effects of either group [F(1, 15) = 1.656, *p* = 0.218] or day [F(2, 30) = 2.125, *p* = 0.137]. Analysis of DI_O_ for WPI alone vs. WPI + Glucose yielded an effect of group for Days 1 (*p* = 0.045) and 7 (*p* = 0.024) but not Day 0 (*p* = 0.147), with CON being higher than PCOS on both days.

### 3.5. Glucagon and Glucagon IAUC

**Figure 8:**
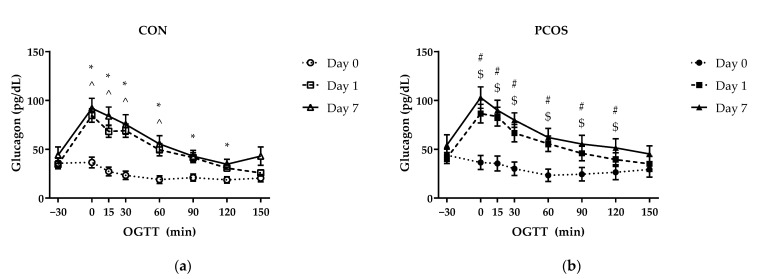
Mean (±SE) glucagon concentrations in (**a**) CON and (**b**) PCOS across timepoints. * = significance between Day 0 and Day 1 for CON; ^ = significance between Day 0 and Day 7 for CON; # = significance between Day 0 and Day 1 for PCOS; $ = significance between Day 0 and Day 7 for PCOS. CON = non-PCOS women; OGTT = oral glycemic tolerance test; PCOS = women with polycystic ovarian syndrome.

Glucagon values across all timepoints and days for both groups are displayed in Figure 8. No interaction effect of “time × day × group” [F(6.46, 168.04) = 1.04, *p* = 0.40], “day × group” [F(1.59, 41.24) = 0.93, *p* = 0.38], “time × day” [F(6.46, 168.04) = 0.86, *p* = 0.53], nor “time × group” [F(3.38, 87.77) = 0.94, *p* = 0.44] were found. A main effect of “day” [F(1.59, 41.24) = 5.17, *p* = 0.02] was present but not for “time” [F(3.38, 87.77) = 2.54, *p* = 0.06]. Additional analyses revealed differences at timepoints within each group between days. For the CON group, Day 0 glucagon levels were lower than Day 1 levels at 0 (*p* < 0.01), 15 (*p* < 0.01), 30 (*p* < 0.01), 60 (*p* < 0.01), 90 (*p* = 0.02), and 120 (*p* = 0.02) min; and Day 0 glucagon levels were lower than Day 7 levels at 0 (*p* < 0.01), 15 (*p* < 0.01), 30 (*p* < 0.01), and 60 (*p* < 0.01) min. For the PCOS group, Day 0 glucagon levels were lower than Day 1 levels at 0 (*p* < 0.01), 15 (*p* < 0.01), 30 (*p* < 0.01), 60 (*p* (0.01), 90 (*p* < 0.01), and 120 (*p* < 0.01) min; and Day 0 glucagon levels were lower than Day 7 levels at 0 (*p* < 0.01), 15 (*p* < 0.01), 30 (*p* < 0.01), 60 (*p* < 0.01), 90 (*p* < 0.01), and 120 (*p* < 0.01) min.

**Figure 9:**
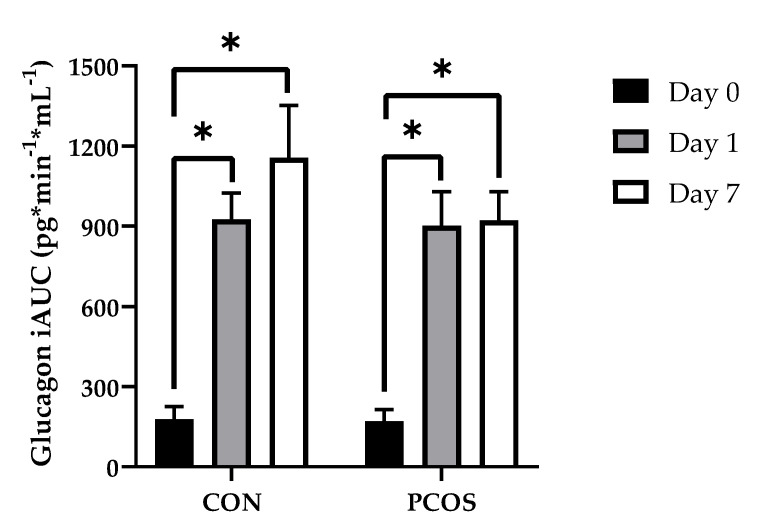
Glucagon iAUC mean values (± SE) in CON (*n* = 14) and PCOS (*n* = 15). * = significance between days, *p* < 0.05. CON = non-PCOS women; iAUC = incremental area under the curve; PCOS = women with polycystic ovarian syndrome.

Figure 9 displays iAUC values for glucagon across all days for both groups. No interaction effect for “day × group” [F(1.62, 42.07) = 1.308, *p* = 0.28] nor main effects for both “day” [F(1.62, 42.07) = 1.95, *p* = 0.16] and “group” [F(1, 26) = 1.24, *p* = 0.28] were found. Post-hoc analyses revealed Day 0 glucagon iAUC to be lower than Day 1 (*p* < 0.01) and Day 7 (*p* < 0.01) in both the CON and PCOS groups.

### 3.6. AST, ALT, and AST:ALT

Table 4 depicts baseline values for AST, ASTact, and ALT across measured days. After adjustment for BF%, AST did not differ among days [F(2, 52) = 0.17, *p* = 0.85] nor between groups [F(1, 26) = 0.06, *p* = 0.81] along with no interaction occurring for day × group [F(2, 52) = 1.32, *p* = 0.28]. ALT also did not differ among days [F(1.52, 39.53) = 0.55, *p* = 0.53] nor between groups [F(1, 26) = 0.00, *p* = 0.99] along with no interaction occurring for “day × group” [F(1.52, 39.53) = 1.06, *p* = 0.34]. Lastly, there was not a significant difference in Day 0 (*p* = 0.52) or Day 7 (*p* = 0.43) ASTact between the groups.

**Figure 10:**
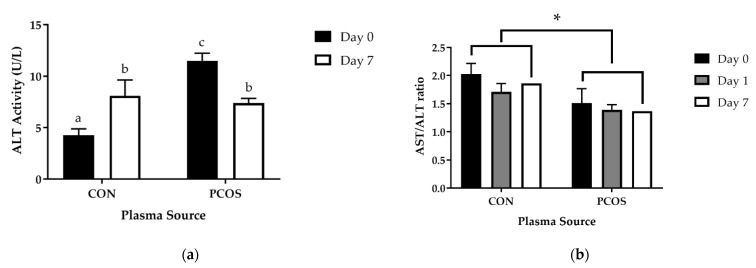
Mean (±SE) (**a**) ALTact and (**b**) AST:ALT levels in CON and PCOS across days different letters equate to statistically significant differences (*p* < 0.05). * = significance between groups, *p* < 0.05. CON = non-PCOS women; OGTT = oral glycemic tolerance test; PCOS = women with polycystic ovarian syndrome.

Figure 10 depicts baseline values for ALTact and AST:ALT across measured days. There was a significant interaction between groups and day on ALT activity (ALTact, Figure 10a) [F(1, 9) = 17.870, *p* = 0.002]. The main effect of group showed a significant difference in mean ALTact on Day 0 [F(1, 10) = 62.826, *p* < 0.0005], but not Day 7 [F(1, 10) = 0.162, *p* = 0.695]. ALTact on Day 0 was significantly greater in PCOS women (11.496 ± 1.806 U/L,) than CON women (4.258 ± 1.547 U/L), *p* < 0.0005. There was a significant effect of day on ALTact for CON participants [F(1, 5) = 7.105, *p* = 0.045]. ALTact on Day 7 was significantly greater in CON women (8.09 ± 3.76 U/L,) than on Day 0, *p* = 0.045. There was also a significant effect of day on ALTact for PCOS participants [F(1, 4) = 13.838, *p* = 0.020]. ALTact on Day 0 was significantly greater in PCOS women than on Day 7 (7.4088 ± 2.767 U/L), *p* = 0.020). There was no significant interaction between groups and day on AST:ALT (Figure 10b) [F(1.44, 54) = 0.428, *p* = 0.0589]. There was a main effect of group [F(1, 27) = 4.449, *p* = 0.044], but not Day F [(1.44, 54) = 0.164, *p* = 0.164]. Post-hoc analysis indicated that AST:ALT was significantly greater in CON women than PCOS women overall.

After adjustment for BF%, there was an effect of day and group on ALTact [F(1, 8) = 10.294, *p* = 0.012]. The main effect of group showed a significant difference in mean ALTact on Day 0 [F(1, 9) = 43.995, *p* < 0.0005], but not Day 7 [F(1, 9) = 0.146, *p* = 0.711]. Day 0 ALTact was significantly greater in PCOS women vs. Control women (mean difference of 6.109 ± 0.921 U/L, *p* <0.0005). Day had an effect on AST:ALT values [F(1.49, 38.81) = 3.74, *p* = 0.04] whereas group did not [F(1, 26) = 0.27, *p* = 0.61]. However, post hoc analyses did not reveal differences among days (*p* > 0.05). No interaction effect was found for day × group on AST:ALT values [F(1.49, 38.81) = 0.64, *p* = 0.49].

### 3.7. SHBG

Table 5 depicts adjusted means and variability for SHBG, TG, TC across measured days with BF% as a covariate. SHBG concentrations were significantly different between groups [F(1, 26) = 4.34, *p* = 0.04]. No differences were reported for day [F(1, 26) = 0.41, *p* = 0.53] nor with the interaction of day × group [F(1, 26) = 2.01, *p* = 0.17]. Post-hoc analyses revealed differences between groups at Day 7 only (*p* = 0.04).

### 3.8. Total Cholesterol and Triglycerides

There was no significant interaction between groups and day on TG concentration [F(1, 10) = 7.09E−06, *p* = 0.998]. The main effect of group had a significant effect on mean TG concentration on the different days [F(1, 10) = 9.756, *p* = 0.011]. Overall, TG concentrations were significantly higher in PCOS women compared to CON women (mean difference = 90.32 SE = 28.917 mg/mL, *p* = 0.011). The main effect of day had no significant effect on mean TG concentration between intervention groups [F(1, 10) = 1.287, *p* = 0.283]. After adjustment for BF%, there were no significant differences in Day 0 (*p* = 0.069) or Day 7 (*p* = 0.120) TG concentrations between the groups (Table 5).

There was not a significant interaction between groups and day on TC [F(1, 11) = 0.098, *p* = 0.76]. Nor was there a main effect of group [F(1, 11) = 4.182, *p* = 0.066] or day [F(1, 11) = 2.81, *p* = 0.122] on TC. After adjustment for BF%, there were no significant differences on Day 0 (*p* = 0.440) or Day 7 (*p* = 0.807) TC concentrations between the groups (Table 5).

### 3.9. Lipid Accumulation

**Figure 11:**
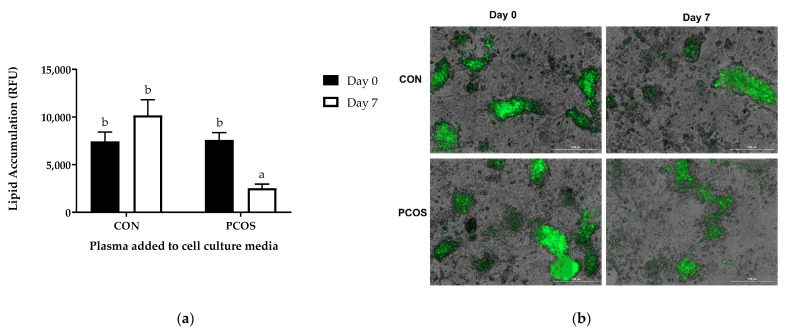
Lipid accumulation in HepG2 cells treated with plasma from CON and PCOS women before and after WPI supplementation: (**a**) bar graph representing the mean  ±  SD of fluorescent levels for each group (CON  =  6, PCOS = 6); (**b**) representative photos of lipid droplets staining (shown in green) by AdipoRed. Different letters equate to statistically significant differences (*p* < 0.05). CON = non-PCOS women; PCOS = women with polycystic ovarian syndrome; RFU = relative florescence units.

Lipid accumulation in HepG2 cells was significantly different between the different plasma sources [F(3, 20) = 7.512, *p* = 0.001] (Figure 11). In the PCOS group, lipid accumulation decreased from baseline (7611.43 ± 3994.3) after 7-days of WP ingestion (2527 ± 1038.19, Figure 11a). Tukey post hoc analysis revealed that PCOS-D7 was significantly lower than CON-D0 (7450.17 ± 2377.18, *p* = 0.032), CON-D7 (10,185 ± 1865.03, *p* = 0.001), and PCOS-D0 (*p* = 0.021), but no other group differences were statistically significant.

## 4. Discussion

The purpose of this exploratory study was to determine the effectiveness of 7-day WPI consumption as a single dose on biological parameters associated with PCOS and NAFLD. The results herein indicate that WPI may be beneficial in reducing some of the negative biological aspects of PCOS and NAFLD. Glucose concentrations were suppressed compared to the normal response throughout most of the OGTT in response to the WPI preload on Days 1 and 7 (Figure 2) in both groups with an associated increase in circulating insulin secretion (Figure 4). Additionally, there was an increase in glucagon secretion in response to the WPI preload during the OGTT in both groups on Days 1 and 7 (Figure 8), an effect that could have occurred to combat falling glucose levels to prevent hypoglycemia. No effect was recorded on iAUC values in response to the WPI 7-day load for glucose (Figure 3), insulin (Figure 5), nor glucagon (Figure 9). Alternatively, SHBG and TG were not affected by a 7-day WPI load (Table 5). While AST and ALT individually were not statistically affected by a 7-day WPI load (Table 4), the AST:ALT was decreased slightly over time (Figure 10b) and ALTact decreased significantly in the PCOS group after WPI preloading (Figure 10a). TC concentrations, while not statistically significant, also decreased in the PCOS group by roughly 13% and the CON group by roughly 8% after 7 days of WP consumption (Table 5). Lastly, lipid accumulation in HepG2 cells was decreased in response to WPI in the PCOS group but not the CON (Figure 11). Based on these findings, our hypothesis for this study can be accepted.

Previous studies have reported the influence of whey protein supplementation on glycemic and liver enzyme parameters in other populations. In healthy young and older adults, 30 g of whey protein resulted in similar responses for insulin and glucagon, with significant increases for both parameters within 30 min of supplementation and a subsequent decrease in concentration over time in addition to increases in AUC values in response to whey protein consumption [50]. Additionally, glucose concentration remained unchanged over time in response to whey protein ingestion, providing further support to the findings in the current study. Similar responses have been reported within obese men for insulin, glucagon, and glucose concentrations in response to whey protein intraduodenal infusion [51]. Alternatively, whey protein is proposed to reduce ALT and AST concentrations after 28 days of supplementation in rats fed a high carb, fat-free diet to induce NAFLD along with improving fatty acid infiltration in hepatocytes and reducing oxidative stress [39]. This is partially consistent from the results of the current study. In our study, the glycemic response appeared to be improved in both the CON and PCOS groups as the peak maximum in response to glucose alone was never matched when whey was consumed and was delayed from 30 min to 120 min in both groups. Whey ingestion resulted in a reduction in the hyperglycemic response to a glucose load, which long-term, may result in better implications for the development of T2DM. The highest AUC for glucose was found following seven days of whey ingestion, indicating that short-term whey ingestion versus a single event does appear to elicit a small but different glycemic response. This could imply that there may be a minor shift in metabolism. It is unknown if these minor shifts in metabolism would have an impact on liver function and stress and fat accumulation. To examine for this possibility, AST and ALT were evaluated as markers of hepatic stress and disease. While AST and ALT levels were unaltered, ALT activity decreased in the PCOS participants. As elevated ALT blood levels is a common index for NAFLD with a AST-to-ALT-ratio (AST:ALT) < 0.8 indicating positive for hepatic steatosis, it is important to assess this parameter when evaluating hepatic stress. Importantly, both ALT activity and levels decreased in women with PCOS resulting in a reduction in the AST-to-ALT ratio. This indicates that whey ingestion in women with PCOS did not increase and may actually reduce the possibility for NAFLD development in PCOS. Thus, while total insulin was increased in response to a whey bolus, it does not appear to lead to increased hepatic stress and the potential for NAFLD. Acute or chronic whey protein supplementation has not been found to have an effect on SHBG concentration [52].

Participants were age-matched with no associated differences in waist-to-hip ratio, height, nor basal insulin sensitivity as indicated by the Matsuda Index (Figure 6). The PCOS group had higher weight (kg), BMI (kg/m^2^), BF%, and LBM (kg) compared to controls (Table 2). AN and HS, hallmarks of the physical attributes of PCOS, were present more often in the PCOS group compared to the CON group. AN was more profound for the severity and texture at the neck. No differences were reported between groups at all other sites (Table 3). This could be due to AN primarily affecting races with a darker skin tone [53,54,55]. Due to the nature of this study and the available population pool, the majority of participants were Caucasian or of Asian descent with few people of color (Table 2). This may help to explain the lower scoring of AN in our study population. Additionally, HS was reported at sites of the upper lip, chin, upper abdomen, and thighs with an overall mFG score higher in the PCOS group.

Obesity and IR are associated with AN and HS. Additionally, hyperandrogenism is associated with HS as a result of low SHBG concentrations with a correlated elevation of free testosterone. Additional data from the current study reported elevated free testosterone levels within the PCOS group at baseline [56], consistent with the physiological properties associated with this condition. Any reduction in body weight, IR, and/or hyperandrogenism may help to alleviate or reduce the severity of these co-morphologies. In this study, insulin sensitivity, as measured by the Matsuda Index (Figure 6), was increased as a result of WPI supplementation for both a single dose and 7-day load. These results infer that chronic WPI supplementation may be a viable option in increasing insulin sensitivity in women with PCOS due to the incretin response imposed by WPI. Previous studies have reported similar insulin sensitivity effects of whey protein involving T2D, overweight, and obese populations [57,58,59,60]. Furthermore, our DI_O_ results, a simple surrogate estimate of β-cell function relative to insulin sensitivity, indicate that WPI alone was able to increase DI_O_ after 7 days of consumption compared to the baseline. However, when the DI_O_ was calculated after both glucose and WPI consumption, there were no significant differences. Overall, our CON participants had higher DI_O_ than our PCOS participants, which is to be expected, given that women with PCOS are at higher risk of T2DM. However, it should be noted that the increase in insulin observed in this short-term study indicates that HS problems could increase with short-term or sporadic whey ingestion.

A recent meta-analysis of 13 randomized controlled trials showed that whey supplementation significantly reduced circulating TG by 0.11 mmol/L but had no effect on TC, low-density cholesterol, or high-density cholesterol [61]. This is different from the current study in which WPI had no effect on TG levels and lowered TC concentrations by 13% in PCOS and 8% in CON (although not significantly, Table 5). One explanation for the difference is the length of the study. The studies analyzed by the meta-analysis ranged from 4 weeks up to 48 weeks, with most of them being 12 weeks long and a daily dose of whey ranging from 0.7 g to 90 g, with the average around 34 g. Our study, however, was only 7 days long, indicating that this short time period might not have been enough to observe differences in TG concentrations after WPI supplementation. It should be noted that while the meta-analysis showed a reduction of TG by 0.11 mmol/L, this may not be clinically relevant compared to TG-lowering medications [61]. While not significant in our study, the decrease seen in TC concentrations could become significant with a longer study and/or additional participants. A recent study was conducted that examined the effects of whey protein vs. simple sugar in women with PCOS over two months. In their study, Kasim-Karakas et al. reported that TC was significantly decreased in the protein group only while TG did not change significantly in either group [62]. This is consistent with the patterns observed in TG and TC concentrations of the current study. Overall, additional studies, with a longer time frame, are still needed to accurately assess the effects of whey supplementation on circulating lipids.

The HepG2 cells indicated a decrease in lipid accumulation in the cells treated with PCOS plasma after WPI supplementation compared to baseline (Figure 11), despite no significant changes in TG or TC levels in the plasma sources (Table 5). This is consistent with previous studies conducted in rodents [63,64]. In a study that evaluated Sprague-Dawley rats fed either casein or whey protein for 2 weeks, there were significant decreases in the expression of several key genes involved hepatic fatty acid synthesis [64]. In obese C57Bl/6J mice fed either an energy-restricted high-fat diet or an energy-restricted whey protein-based high-calcium diet, Pilvi et al. identified a reduction in hepatic lipid accumulation and lipid droplet size in both energy-restricted diets but only the whey protein-based high-calcium diet was able to significantly decreased blood glucose and serum insulin [63]. Additional studies, especially those conducted in humans, are needed to fully understand the effects of whey supplementation on lipid accumulation in the liver.

One limitation to this study is the disproportionate inclusion of women who were considered overweight or obese in the PCOS group. Approximately 71% of the PCOS participants were considered overweight or obese as compared to only 20% of the CON participants. BMI-matched participants were unattainable due to the nature of this study and the available PCOS and CON populations. Because of this, BF% was used as a covariate for analyses when appropriate. A second limitation to this study is the short supplementation timeframe of WPI within this population. Current studies being conducted in our laboratory include WPI supplementation over the course of 40 days to examine the effects of a longer-term supplementation on inflammation, glycemic control, liver enzymes, and androgen hormones within the PCOS population. A third limitation is although total caloric and macronutrient consumption was recorded by participants for the 3 days prior to the start of the study, the overall amino acid composition was not recorded. Given that whey protein is high in branched-chain amino acids, which have been reported to lead to the loss of glycemic control, it is possible that the effects seen in this study are only acute, and long-term supplementation may be detrimental to women with PCOS and NAFLD development. Another limitation is the small sample size of the study (29 participants total), which might have decreased the statistical power of our results. Lastly, the lack of randomization and control supplements could be a limitation. Given that the main focus of this exploratory study was to evaluate the differing effects of WPI on women with and without PCOS, a control supplement was not added to the study.

## 5. Conclusions

This exploratory study serves as a novel and potentially therapeutic use of acute and short-term WPI supplementation to alleviate physiological morphologies in women who are overweight or obese and are diagnosed with PCOS. With the recognized changes in the lipid accumulation of HepG2 cells and plasma glucose levels in the present study and the indication that a longer period of whey protein supplementation may reduce TC, ALT and AST, WPI may be an effective means of reducing NAFLD in women with PCOS. It is intended that our 40-day study may shed more light on the impact of WPI supplementation on parameters associated with NAFLD.

## Figures and Tables

**Table 1:** Self- reported macronutrient and total calorie consumption in PCOS and CON groups at baseline.

	CON Mean ± S.E.	PCOS Mean ± S.E.	*p*-Value
Total calorie intake (kcal/d)	1616.6 ± 83.4	1705.9 ± 127.5	0.56
Carbohydrate intake (g/d)	192.3 ± 11.4	212.7 ± 15.4	0.29
Fat intake (g/d)	61.8 ± 4.4	68.6 ± 7.3	0.42
Protein intake (g/d)	60.9 ± 4.1	57.1 ± 5.0	0.56

Data are expressed as mean *±* S.E. Values are baseline measurements only. Student’s two-tailed *t*-test were used to compare baseline variables in both the CON and PCOS group. There were significant differences between groups for either total calorie consumption or macronutrient consumption. CON = control group; PCOS = polycystic ovary syndrome group.

**Table 2:** Participant characteristics.

	CON Mean ± S.E.	PCOS Mean ± S.E.	*p*-Value
Age (year)	21.1 ± 0.8	22.9 ± 1.6	0.31
Weight (kg) *	63.5 ± 2.4	92.2 ± 7.1	<0.01
Height (cm)	161.3 ± 1.9	165.0 ± 2.2	0.29
BMI (kg/m^2^) *	24.4 ± 1.0	33.7 ± 2.5	<0.01
WHR	0.8 ± 0.0	0.9 ± 0.0	0.48
BF% *	38.4 ± 1.5	46.2 ± 2.0	<0.01
LBM (kg) *	24.7 ± 1.7	44.3 ± 5.0	<0.01
Ethnicity:			
Asian	7 (47%)	2 (14%)	
Caucasian	2 (13%)	9 (64%)	
Hispanic	3 (20%)	3 (21%)	
Other	3 (20%)		

Data are expressed as mean *±* S.E. Values are baseline measurements only. Ethnicity is expressed as number of participants with percentage in parenthesis. A multivariate analysis was used to distinguish differences between groups for participant characteristic data. BF% = body fat percent; BMI = body mass index; CON = control group; LBM = lean body mass; PCOS = polycystic ovary syndrome group; WHR = waist-to-hip ratio. * = Significance, *p* < 0.05.

**Table 3:** AN and hirsutism characteristics.

	CON Mean ± SE	PCOS Mean ± SE	*P*-Value
AN: Severity at neck *	0.07 ± 0.07	1.21 ± 0.39	<0.01
AN: Texture at neck *	0.07 ± 0.07	1.00 ± 0.30	<0.01
AN: Severity at axillae	0.60 ± 0.25	1.29 ± 0.35	0.12
AN: Knuckles	0.07 ± 0.07	0.29 ± 0.13	0.13
AN: Elbows	0.07 ± 0.07	0.21 ± 0.11	0.27
AN: Knees	0.07 ± 0.07	0.14 ± 0.10	0.52
HS: Upper lip *	0.07 ± 0.07	0.86 ± 0.31	0.02
HS: Chin *	0.07 ± 0.07	0.86 ± 0.31	0.02
HS: Chest	0.40 ± 0.24	0.71 ± 0.24	0.36
HS: Upper back	0.07 ± 0.07	0.14 ± 0.10	0.52
HS: Lower back	0.13 ± 0.13	0.64 ± 0.25	0.08
HS: Upper arm	0.20 ± 0.14	0.07 ± 0.07	0.44
HS: Upper abdomen *	0.20 ± 0.11	0.93 ± 0.30	0.03
HS: Lower abdomen	0.33 ± 0.13	0.64 ± 0.25	0.27
HS: Thighs *	0.53 ± 0.22	1.21 ± 0.24	0.04
HS: Total mFG score *	2.00 ± 0.83	6.07 ± 1.75	0.04

Data are expressed as mean *±* SE. Values are baseline measurements only. A multivariate analysis was used to distinguish differences between groups for participant characteristic data. AN = acanthosis; CON = control group; HS = hirsutism; mFG = modified Ferriman Galleway; PCOS = polycystic ovary syndrome group. * = Significance, *p* < 0.05.

**Table 4:** Adjusted means and variability for AST, AST activity, and ALT.

	CON Mean ± SE	PCOS Mean ± SE	*P*-Value
**AST**			0.81
Day 0	23.6 ± 1.2	25.2 ± 1.3	0.42
Day 1	24.8 ± 1.8	23.9 ± 1.9	0.76
Day 7	25.4 ±1.9	23.1 ±2.0	0.44
**ASTact**			0.85
Day 0	5.4 ± 1.9	5.3 ± 1.4	0.52
Day 7	5.3 ± 1.4	7.6 ± 1.7	0.43
**ALT**			0.99
Day 0	15.3 ± 1.8	17.5 ± 1.9	0.44
Day 1	18.3 ± 2.4	16.6 ± 2.5	0.65
Day 7	16.8 ±1.7	16.3 ±1.8	0.85

Values are baseline, Day 1, and Day 7 measurements. An analysis of covariance with body fat percentage used as the covariate was used to distinguish differences between CON (*n* = 15 for ALT, and AST; *n* = 6 for ASTact) and PCOS (*n* = 14 for ALT, and AST; *n* = 6 for ASTact). No significant differences were observed between groups or across the days for each. ALT = alanine aminotransferase; AST = aspartate aminotransferase; ASTact = aspartate aminotransferase activity; CON = control group; PCOS = polycystic ovary syndrome group.

**Table 5:** Adjusted means and variability for SHBG, TG, and TC, with body fat percentage as a covariate.

	CON Mean ± SE	PCOS Mean ± SE	*p*-Value
**SHBG** ^a^			0.047
Day 0	38.2 ± 15.3	83.1 ± 15.9	0.07
Day 7 ^b^	29.2 ± 19.7	94.2 ± 20.5	0.043
**TG**			0.08
Day 0	112.2 ± 16.9	162.4 ± 15.4	0.07
Day 7	123.9 ± 20.5	134.6 ± 16.4	0.73
**TC**			0.44
Day 0	186.1 ± 13.2	170.0 ± 12.0	0.44
Day 7	161.2 ± 9.8	157.7 ± 8.9	0.81

Values are baseline, Day 1, and Day 7 measurements. Analysis of covariance with body fat percentage used as the covariate was used to distinguish differences between CON (*n* = 15 for SHBG; *n* = 6 for TG and TC) and PCOS (*n* = 14 for SHBG; *n* = 6 for TG and TC). CON = control group; PCOS = polycystic ovary syndrome group; SHBG = sex-hormone binding globulin; TC = total cholesterol; TG = triglycerides. a = significance between groups (*p* < 0.05). b = significant between groups within the respective day (*p* < 0.05).

## Data Availability

The datasets used and/or analyzed during the current study are available from the corresponding author on reasonable request.
